# Teenage pregnancy: focus on people with mental disorders

**DOI:** 10.3389/fpsyt.2024.1305572

**Published:** 2024-02-02

**Authors:** Zhiwei Liu, Liang Sun, Rongchun Yang, Shu Cui, Gaofeng Yao, Yun Liu, Huanzhong Liu

**Affiliations:** ^1^ Department of Psychiatry, The Third People’s Hospital of Fuyang, Fuyang, China; ^2^ Department of Psychiatry, Chaohu Hospital of Anhui Medical University, Hefei, China; ^3^ School of Mental Health and Psychological Sciences, Anhui Medical University, Hefei, China

**Keywords:** pregnancy, mental disorders, pregnancy complications, adolescence, proposal

## Introduction

Teenage pregnancy is a global phenomenon caused by multiple and complex factors that can have serious health, social and economic consequences. It was reported in 2020 that there are about 21 million pregnancies per year among girls aged 15-19 in low - and middle-income countries, about 50% of which are unintended, and about 12 million end up giving birth (1). Globally, the adolescent birth rate declined from 64.5 per 1,000 women in 2000 to 42.5 per 1,000 women in 2021 (2). Despite the global decline in adolescent fertility, the rate of change is uneven across regions and there are large differences in fertility between countries and across regions within countries. A study in the United States population noted that adolescent pregnancy accounts for approximately 6% of the maternal population and is higher among those with lower education or lower economic status (3). In China, a large sample survey noted that teenage pregnancies accounted for 1.54% of maternal pregnancies under the age of 35, which is significantly lower than in Western countries (4). And the relaxation of China’s one-child policy may also have had an impact on teenage pregnancy. Early pregnancy and childbearing increase the risk to both the mother and her newborn. Compared to young adult pregnant women, teenage mothers are at higher risk of abortion, stillbirth, eclampsia, puerperal endometritis and systemic infections, and babies born to teenage mothers are at higher risk of low birth weight, preterm birth and severe neonatal diseases. While some progress has been made worldwide in reducing adolescent first birth rates, progress has been slower among adolescents with mental disorders and other vulnerable groups, contributing to growing inequalities (5, 6). This article focuses on the risks that teenage pregnancy poses to health and social outcomes, especially for adolescents with mental health problems. It is also noted that current prevention measures are often insufficient to provide the necessary care to vulnerable adolescents. At the end, we conclude by drawing on observations and evidence from global sources to support a robust set of nationally-bound proposals in China.

## Mortality rate in teenage pregnancy

The Global Adolescent Mortality Report noted that despite a rapid decline in maternal deaths as a proportion of total deaths, complications from pregnancy and childbirth remain among the leading causes of death among girls aged 15-19 years globally (7), and results obtained in the Chinese adolescent population are consistent with this (8). WHO data stated that the leading causes of maternal death were hemorrhage (27.1%), hypertensive disorders (14.0%) and sepsis (10.7%), which accounted for more than half of all causes of death (9). In the China Population Survey, adolescent maternal mortality rates have generally declined rapidly across Chinese provinces, with the adolescent maternal mortality ratio (MMR; number of maternal deaths per 100 000 livebirths) for 10-14 year old declining from 76.4 (66.1-88.3) in 1990 to 6.8 (6.0-7.7) in 2017, and from 1465.3 (1270.3-1675) in 1990 to 69.8 (60.7-81.5) in 2017 for adolescent maternal deaths aged 15-19 years, with hemorrhage and hypertensive disorders being the two leading causes of maternal mortality (10). A Canadian study noted that maternal age 19 years and younger was an independent influencing factor for severe maternal morbidity (11). Compared to mothers aged 20-34 years, mothers aged 10-19 years had a higher risk of preterm delivery, stillbirth, small for gestational age, and neonatal death, while the risk of cesarean delivery and gestational diabetes was lower (4).

## Stillbirth in teenage pregnancy

The higher rate of stillbirths in teenage pregnancies is likewise a problem that cannot be ignored. Stillbirth rates are significantly higher for adolescent pregnancies among 10–19-year-olds compared to adult pregnancies (4). A recent systematic review reported that stillbirth rates have declined in 114 countries worldwide since 2000, but progress in reducing them has been slow, with significant pre-country disparities in stillbirth prevention, particularly in low- and middle-income countries (12). The survey indicated that maternal mortality rates in Africa have declined from previous levels, but only North Africa is close to the UN Sustainable Development Goal that the global maternal mortality ratio should be less than 70 deaths per 100,000 live births (13). A former research covering eight countries in South Asia also showed that adolescent maternal fertility and neonatal mortality rates are higher and significantly associated with local health care payment (14). Higher stillbirth rates in adolescent pregnancies can place a considerable burden on their health, families and society.

## Teenage pregnancy with mental disorders

Teenage pregnancy is gaining attention and focus, but little is being mentioned about the current status of teenage pregnancy with mental disorders. The sexual and reproductive health of adolescent psychiatric patients has been largely overlooked. A large sample study in Canada noted that girls with severe mental disorders had significantly smaller decreases in fertility compared to adolescents without mental disorders (5). However, adolescents with mental disorders were more likely to experience risky sexual behavior and were at higher risk for sexually transmitted infections than adolescents without mental disorders (6). One study noted that 26.3% of women with mental disorders aged 15-25 years had been pregnant, 95.2% of which were unintended, while 25.5% tested positive for sexually transmitted infections (STI) (15). In another small sample study, it was found that 78.3% of adolescents with mental disorders had ever had sex, 22.5% had been previously pregnant, and 18.6% tested positive for STI (16). Furthermore, women with mental illness are more likely to have recurrent abortions, sexually transmitted infections, cancers of the reproductive system, contraception and emergency contraception than women without mental illness (17). Current evidence indicated that the rate of pregnancy and STI infection seems to be higher among adolescent girls with mental disorders, which seriously affects their own health.

## Associated and postoperative pregnancy complications

In terms of pregnancy complications, women with mental disorders are more likely to have recurrent abortions, pregnancy terminations, gynecological disorders, sexually transmitted infections, and reproductive cancers. However, the sexual and reproductive health needs of women with mental disorders are far from being met compared to the healthy female population, constituting a serious health inequality (17). In terms of specific diseases, pregnancies in schizophrenic population had a higher risk of maternal pre-eclampsia, gestational hypertension, gestational diabetes (18), preterm birth, stillbirth, infant mortality, maternal metabolic risk (19), venous thromboembolism, abnormal birth weight size for gestational age (20), and a higher incidence of neonatal morbidity. For this population, there might be a need for more intensive care hospital resources, more meticulous perinatal care and more complex interventions (20). Secondly, miscarriage and abortion rates were significantly higher in women with schizophrenia, especially in patients who are young (<25 years), have multiple births, and have a history of prior substance abuse (21). On the other hand, maternal major depression was associated with preterm birth and small for severe gestational age; maternal bipolar disorder was associated with preterm birth and large for severe gestational age, and both mood disorders were correlated with higher risk of congenital malformations, neonatal morbidity, and neonatal readmission (22). Meta-analysis also noted that maternal risk of stillbirth or neonatal death was significantly higher in mothers with depression, anxiety, or severe mental disorders (23).

Despite strong evidence of significantly higher maternal pregnancy and delivery complications for women with mental disorders, related interventions are progressing more slowly and the sexual and reproductive health needs of these vulnerable populations are difficult to secure. In addition, the information available on adolescent pregnancy with mental disorders is extremely limited. The limited evidence points to a significantly higher incidence of teenage pregnancy in mental disorders, with roughly one in five to one in three patients having had an unplanned pregnancy (15, 16, 24). Moreover, children of teenage mothers were more likely to be affected by their mothers’ psychiatric disorders, making them a high-risk group for mental disorders (25). In addition, the high rate of unplanned pregnancies observed in adolescents also suggests that teratogenicity should be considered when prescribing psychotropic drugs to adolescent girls, as exposure to psychotropic drugs in early pregnancy may harm the developing fetus, and often the fact of pregnancy is difficult to detect in time in adolescents with unplanned pregnancies.

## Factors associated with teenage pregnancy

There are numerous factors associated with adolescent pregnancy, such as early marriage, risky sexual behavior, substance use, family experiences of adolescent childbearing, and lack of sex education and health services. Early marriage is one of the main causes of teenage pregnancy, as it is difficult for girls who marry early to make their own choices about delaying childbirth or using contraceptives, which in turn has a negative impact on the health of children who marry at an early age and their children. Studies have shown that offspring of teenage mothers were also more likely to become teenage parents and that offspring of teenage mothers were prone to poorer educational achievement and lower life satisfaction, resulting in socially and economically risky generational chains (26). The Guttmacher Institute survey have revealed that the higher teenage pregnancy rates tend to be related with lower educational attainment or lower economic status (27). Otherwise, previous study had shown that the prevalence of disability was significantly higher among men and women who married as children, especially among those who married under the age of 16 (28). In addition, WHO reported that sexual abuse against children increases the risk of unintended pregnancy and that at least one in eight children in the world has been sexually abused before 18 years old (29). Despite the current marked decline in fertility among girls aged 15-19, WHO and related agencies survey shown that little progress has been made in preventing adolescent pregnancy, abortion, maternal mortality, sexually transmitted infections, and HIV, particularly among the mentally challenged adolescent population (2, 5, 6).

## Gaps in data on teenage pregnancy with mental disorders in China

In China, there is a significant lack of research on teenage pregnancy with mental disorders, resulting in gaps in data and significant disparities across the entire continuum of family, school, community, social, legal, and official policies. Adolescents need and have the right to comprehensive sexuality education and easier access to contraceptive information and services, and the number of early pregnancies and births among girls can be reduced by measures such as a minimum age for marriage. At the same time, girls with mental disabilities who become pregnant need access to quality prenatal care. The “Central Subsidy for Local Health Funding for the Management and Treatment of Serious Mental disorders Project”, commissioned by the Department of Disease Control of the Ministry of Public Health of China in 2004 and undertaken by the Mental Health Center of the Chinese CDC, is a disease management project for patients with serious mental disorders. Its purpose is to strengthen mental health construction and other aspects, specifically: (1) registration and assessment of patients with severe mental disorders, and follow-up of patients with risky behavioral tendencies; (2) provision of free primary medication for mental disorders to poor patients with risky behavioral tendencies; free laboratory tests; emergency treatment of patients; free emergency hospitalization; unlocking and treatment of locked patients (3); management and treatment of severe mental disorders program Training of relevant personnel. From the specific inclusion of information entries, it is clear that for patients with severe mental disorders, their pregnancy is not included in the content of the disease management survey (30), while for adolescent patients with severe mental disorders most provinces and municipalities adopt a natural strategy of not recording and following up, and knowledge about the management of this population’s condition or pregnancy is even more lacking.

## Proposals

There should be several initiatives for teenage pregnancy with mental disorders. First, families and schools should strengthen education on contraception and reproductive health for adolescents to reduce the rate of unprotected sex and unwanted pregnancies. Adolescents have a significantly higher risk of pregnancy mortality and complications than adults, but access to health care for adolescent mothers is relatively limited, especially among those with mental disorders. Therefore, the provision of counseling and perinatal health services by health care professionals to adolescent primigravida and their families with flexible and appropriate perinatal health programs based on adolescent primigravida risk factors will help improve the health of adolescent primigravida and their infants. According to studies, increasing health care expenditures and reducing adolescent female fertility may contribute to a reduction in maternal and neonatal mortality (14). There is a need to focus on improving the education of adolescents and removing economic barriers that prevent them from accessing maternal health services (31). Second, diverse public health facilities and community-based adolescent parenting and child care programs, such as breastfeeding, economic, parenting, and nutritional support, should be established, improved, but this link is currently quite weak, and those pregnant women with severe mental disorders have difficulty participating in and accessing these simple, decentralized resources. However, community-based psychiatric rehabilitation services have largely come to a complete halt since the outbreak of the COVID-19 epidemic, and community-based rehabilitation for mental health in the post-epidemic era is at a new beginning. Third, the law should focus on protecting the personal rights of underage mothers, strictly reduce and control the incidence of sexual assault on the underage population, take legal action or hold the assailants legally responsible, provide timely medical care and psychological support to victims and related witnesses, and do its best to alleviate their physical and psychological trauma. Moreover, in order to further reduce the sexual abuse suffered by minors, in May 2020, the Supreme People’s Procuratorate of the People’s Republic of China, the Ministry of Education, and other departments jointly issued the Opinions on Establishing a Compulsory Reporting System for Cases of Aggression against Minors (for Trial Implementation) to establish a compulsory reporting system for cases of aggression against minors. According to the national law, when a pregnant girl under 14 years of age is registered to the hospital, the hospital will compulsorily report to the public security authorities or the procuratorial authorities if the medical staff is confronted with possible aggression against minors and the risk of wrongful aggression (32). Minors are theoretically going to hospitals after being assaulted, and hospitals and doctors often serve as the first to know about minors being assaulted, and the implementation of a mandatory reporting system in this part of the hospital can directly determine the effectiveness of the system’s implementation. This reporting system is a major measure of national protection for adolescents. At present, it is still being gradually promoted, and it is worth further deepening the understanding and practical application of the general population and professionals. Lastly, at the national level, the disease management system for people with mental disorders should be further improved to provide more health care services and interventions for people with perinatal mental disorders in order to promote basic reproductive health for the population at large. The health care system should promote further investigation of the current status of teenage pregnancies with mental disorders to obtain relatively credible and realistic data, which in turn can identify creative solutions to provide better clinical care and medical services for this vulnerable population.

## Author contributions

ZL: Writing – original draft, Writing – review & editing. SL: Investigation, Software, Writing – review & editing. RY: Data curation, Investigation, Writing – original draft, Writing – review & editing. SC: Conceptualization, Formal Analysis, Writing – original draft, Writing – review & editing. GY: Data curation, Investigation, Software, Writing – original draft. YL: Data curation, Methodology, Writing – review & editing. HL: Project administration, Supervision, Writing – original draft, Writing – review & editing.
